# Effects of Cognitive Behavioral Therapy-Based Educational Intervention Addressing Fine Particulate Matter Exposure on the Mental Health of Elementary School Children

**DOI:** 10.3390/children12081015

**Published:** 2025-08-01

**Authors:** Eun-Ju Bae, Seobaek Cha, Dong-Wook Lee, Hwan-Cheol Kim, Jiho Lee, Myung-Sook Park, Woo-Jin Kim, Sumi Chae, Jong-Hun Kim, Young Lim Lee, Myung Ho Lim

**Affiliations:** 1 Department of Psychology, Graduate School, Dankook University, Cheonan 31116, Republic of Korea; zzazzacnamu@dankook.ac.kr (E.-J.B.); 72241478cha@dankook.ac.kr (S.C.); 2 Department of Occupationl and Environmental Medicine, Inha University Hospital, Inha University, Incheon 22332, Republic of Korea; taesanglee89@inha.ac.kr (D.-W.L.); carpediem@inha.ac.kr (H.-C.K.); 3 Department of Occupationl and Environmental Medicine, Ulsan University Hospital, Ulsan University, Ulsan 44033, Republic of Korea; leejh@uuh.ulsan.kr; 4 Institute of Environmental Medicine, Seoul National University Medical Research Center, Seoul 03080, Republic of Korea; snupms@snu.ac.kr; 5 Department of Internal Medicine, College of Medicine, Kangwon National University, Chuncheon 24341, Republic of Korea; pulmo2@kangwon.ac.kr; 6 Department of Healthcare Policy Research, Korea Institute for Health and Social Affairs, Sejong 30147, Republic of Korea; csm1030@kihasa.re.kr; 7 Department of Social and Preventive Medicine, School of Medicine, Sungkyunkwan University, Suwon 16419, Republic of Korea; kimjh32@skku.edu; 8 Department of Psychology and Psychotherapy, College of Health Science, Dankook University, 119 Dandae-ro, Dongnam-gu, Cheonan 31116, Republic of Korea

**Keywords:** cognitive behavioral therapy, PM2.5, environmental health education, elementary school children, anxiety

## Abstract

**Objectives**: This study assessed the effectiveness of a cognitive behavioral therapy (CBT)-based fine dust education program, grounded in the Health Belief Model (HBM), on elementary students’ fine dust knowledge, related behaviors, and mental health (depression, anxiety, stress, sleep quality). **Methods**: From September to November 2024, 95 students (grades 4–6) living near a coal-fired power plant in midwestern South Korea were assigned to either an intervention group (n = 44) or a control group (n = 51). The intervention group completed a three-session CBT-based education program; the control group received stress management education. Assessments were conducted at weeks 1, 2, 4, and 8 using standardized mental health and behavior scales (PHQ: Patient Health Questionnaire, GAD: Generalized Anxiety Disorder Assessment, PSS: Perceived Stress Scale, ISI: Insomnia Severity Index). **Results**: A chi-square test was conducted to compare pre- and post-test changes in knowledge and behavior related to PM2.5. The intervention group showed significant improvements in seven fine dust-related knowledge and behavior items (e.g., PM2.5 awareness rose from 33.3% to 75.0%; *p* < 0.05). The control group showed limited gains. Regarding mental health, based on a mixed-design ANCOVA, anxiety scores significantly declined over time in the intervention group, with group and interaction effects also significant (*p* < 0.05). Depression scores showed time effects, but group and interaction effects were not significant. No significant changes were observed for stress, sleep, or group × PM2.5 interactions. **Conclusions**: The CBT-based education program effectively enhanced fine dust knowledge, health behaviors, and reduced anxiety among students. It presents a promising, evidence-based strategy to promote environmental and mental health in school-aged children.

## 1. Introduction

Fine dust (or fine particulate matter, PM2.5) is one of the six standard air pollutants monitored by the US Environmental Protection Agency (EPA). These pollutants, which include ozone, nitrogen oxides, sulfur dioxide, lead, and carbon monoxide, are all considered potential health hazards to humans (EPA, 2021) [1]. According to the World Health Organization’s 2018 data, fine particulate matter with a diameter of less than 2.5 mm (PM2.5) is responsible for approximately 800,000 premature deaths worldwide and accounts of for 40% of all deaths related to outdoor air pollution [2]. PM2.5 is small enough to penetrate deep into the lower respiratory tract and can pass through the blood–brain barrier, potentially causing systemic inflammation and oxidative stress, which can ultimately result in neurotoxic effects on the brain [3]. Chronic exposure to such neurotoxins can lead to structural and functional changes in the brain, resulting in a range of cognitive, behavioral, and emotional problems [4,5]. Numerous studies have documented the adverse effects of air pollution on mental health outcomes such as depression, anxiety, and suicidal behavior, particularly in adults [6,7,8]. Children and adolescents are particularly vulnerable due to their ongoing brain development [9]. Once damage to the nervous system occurs during this critical period, the aftereffects can affect them for a lifetime [10]. According to a report by the World Health Organization (WHO) [11,12], approximately 93% of children under the age of 15 worldwide are exposed to polluted air, posing significant risks to their health and development. Therefore, investigating the impact of air pollution on emotional and mental health disorders in children and adolescents is both important and urgent [13].

Three meta-analyses targeting children and adolescents have identified an association between exposure to PM2.5 and depression [14,15,16], and a cohort study conducted in the UK revealed that exposure to PM2.5 and nitrogen oxides significantly increased the risk of developing a major depressive disorder by age 18 [17]. Two separate cohort studies have also reported a significant association between fine dust exposure and anxiety [18,19]. Regarding the effects of air pollution on sleep disorders in children, Lawrence et al. [20] found that exposure to PM1 was associated with the highest odds of sleep disorders, while Abou-Khadra et al. [21] reported a strong link between PM10 exposure and sleep disorders.

Although the Korean government has recommended various preventive measures to reduce health risks from fine dust exposure, such as limiting outdoor activities, wearing protective masks, and using air purifiers to improve indoor air quality, there is still a lack of intervention studies aimed at mitigating the health impacts of PM exposure [22]. To date, most interventions have focused on air purifiers [23], masks [24], pharmaceuticals and dietary strategies [25], and national and community-based studies [26], while behavioral interventions have been significantly under-researched. Among the studies on behavioral interventions, one involving patients with chronic obstructive pulmonary disease (COPD) found that education on self-care led to a reduction in exposure to fine dust and improvements in certain symptoms [27]. Similarly, a study targeting pregnant women demonstrated that an intervention providing information via text messages, including air pollution indices, location data, and exposure duration, resulted in significant improvements in perceived severity, preventive behaviors, and self-efficacy [28,29]. While many studies have also highlighted the mental health risks associated with fine dust exposure in vulnerable populations, such as children and adolescents, there remains a lack of research demonstrating the effectiveness of CBT-based intervention programs designed to facilitate cognitive shifts through various solution-focused and goal-oriented methods.

In response to this need, this study implemented a CBT program based on the Health Belief Model (HBM). The HBM suggests that health-related behaviors are influenced by various factors, including perceived susceptibility, perceived severity, perceived benefits, perceived barriers, cues to action, and self-efficacy [30]. Furthermore, Beck’s cognitive theory (1979) indicates that psychological disorders (such as anxiety, depression, somatic symptoms, and substance abuse) can be alleviated by correcting maladaptive cognitions, such as negative automatic thoughts and dysfunctional attitudes, particularly in stressful situations [31,32,33]. Recent research indicates that cognitive behavioral therapy (CBT) supports health-protective behaviors in children and adolescents by targeting maladaptive cognitions and enhancing self-efficacy [34,35]. For example, CBT interventions help youth recognize and modify negative thought patterns, regulate emotional responses, and develop adaptive coping strategies in response to environmental and psychological stressors. Furthermore, increased self-efficacy through CBT has been shown to reduce anxiety symptoms and improve social functioning in children [36]. Therefore, this research developed an intervention program that sought to enhance general knowledge about fine dust and aimed to mitigate mental health problems caused by negative emotions, including depressive symptoms.

In this study, the intervention was developed based on each component of the Health Belief Model (perceived susceptibility, perceived severity, perceived benefits, perceived barriers, cues to action, and self-efficacy). For example, children were guided to recognize that fine dust can impact their health and were informed about the potentially serious consequences, such as various physical illnesses and influences on mental health. The benefits of wearing masks when going outdoors and using air purifiers were explained, and efforts were made to ensure that a lack of information would not become a barrier to healthy behaviors. In particular, the educational program utilized various visual materials, game elements, and practical activities to foster children’s self-efficacy—that is, the belief that ‘my actions can reduce the negative effects associated with fine dust’—and created an environment that encouraged motivation for action. Ultimately, this study aimed to go beyond mere knowledge transmission, seeking to have students find solutions to the problems they face themselves and to promote behavioral change. Therefore, this study aimed to promote coping behaviors related to fine dust exposure and to confirm their effectiveness, based on the Health Belief Model and cognitive behavioral therapy.

## 2. Methods

### 2.1. Study Design

A CBT-based participatory intervention program designed to improve general knowledge about fine dust and promote mental health was developed and implemented for elementary school students living near a coal-fired power plant. Participants were divided into an intervention group and a control group. This study employed a cluster randomized controlled trial (cluster RCT) design. Classes (clusters) of 4th to 6th grade students were randomly assigned to either the intervention group or the control group. The intervention group participated in three sessions of the intervention program during weeks 1, 2, and 4, with the control group receiving three stress management sessions during the same period. Evaluations of the program’s effectiveness were conducted at weeks 1, 2, 4, and 8.

### 2.2. Participants

The study included students in grades 4–6 from an elementary school recruited using volunteer sampling. Participants were divided into an intervention group (50) and a control group (60). Only the students who attended all sessions were included in the final analysis, resulting in the exclusion of 6 students from the intervention group and 9 from the control group. Consequently, the final analysis included 44 students from the intervention group and 51 students from the control group. The intervention program was conducted from September to November 2024. The CBT-based participatory intervention focused on discussion and active participation rather than passive knowledge delivery. Its primary goal was to promote behavioral change through behavioral interventions, with children actively involved as the main agents in providing information.

### 2.3. Intervention

The program in this study was a CBT-based participatory behavioral change intervention designed to improve children’s general knowledge about fine dust and, consequently, reduce mental health risk factors. The intervention aimed to enhance general knowledge about fine particulate matter, improve children’s health behaviors, and alleviate symptoms of depression, anxiety, stress, and sleep problems. Ultimately, this study sought to develop an intervention that could be directly implemented by professionals in mental health and welfare institutions, as well as by teachers working with children. The intervention group program was led by three Ph.D. candidates in psychology and one master’s student in counseling psychology. The control group program was led by one master’s student in counseling psychology and one Ph.D. candidate in psychology. All sessions were conducted under the guidance and supervision of a licensed psychiatrist.

The program consisted of three 40 min sessions, which were held in weeks 1, 2, and 4. During the CBT-based sessions, children were instructed to close their eyes and imagine how they would behave in situations with high levels of fine dust (imagery), to quietly repeat to themselves the actions they should take (self-instruction), and to write down what they need to do (self-monitoring).

Specifically, the children learned about the definition and size of fine dust (PM2.5), its sources, the dangers it poses, its impact on mental health, how to check PM2.5 levels, and ways to reduce exposure. Preventive actions taught included keeping windows closed at school and at home, ventilating occasionally by opening windows or using exhaust fans, avoiding roads with heavy traffic, washing hands, drinking water, taking vitamin C, eating vegetables, using wet mops instead of vacuum cleaners on days with high fine dust levels, and wearing masks.

The educational methods combined breathing relaxation exercises led by the facilitator, psychoeducational approaches to deliver information, worksheets, and the jigsaw cooperative learning method, which involves children teaching and learning from one another. Each session included homework assignments to help children practice and master coping behaviors related to fine dust exposure.

### 2.4. Measures

#### 2.4.1. Air Quality Monitoring System

The IAQ-CW (LTE) model from Kweather (which employs the light scattering method) was used to measure fine particulate matter. These monitoring devices (which measure PM2.5, PM10, temperature, humidity, CO_2_, VOCs, and noise levels) were installed in each classroom where the behavioral intervention was conducted, as well as in the cafeteria and after-school classrooms. Measurements were taken every second and updated every minute.

#### 2.4.2. Patient Health Questionnaire (PHQ)

The PHQ-9 is a self-report questionnaire developed by Kroenke et al. [37] to assess the severity of depression. It consists of 9 items that align with the DSM-IV diagnostic criteria for major depressive disorder. Each question asks about the frequency of a specific depressive symptom over the past two weeks, using a 4-point Likert scale ranging from 0 (not at all) to 3 (nearly every day). Due to its brief testing time, high sensitivity and specificity for depression, the PHQ-9 is widely used for mental health assessments for various medical conditions. The total score ranges from 0 to 27, obtained by summing the scores of all items. Scores are interpreted as follows: a score of 0–4 points indicates no or minimal depression, 5–9 points indicates mild depression, 10–14 points indicates moderate depression, 15–19 points indicates moderately severe depression, and 20–27 points indicates severe depression. In this study, the version validated by Ahn et al. [38] was used, with a Cronbach’s α of 0.95. The Cronbach’s α for this study was 0.75. All psychological measures were administered using validated Korean-language versions.

#### 2.4.3. Generalized Anxiety Disorder Assessment (GAD)

The GAD-7 is a self-administered questionnaire used to screen for and assess the severity of anxiety. It consists of 7 items and is known for its high reliability and ease of use, making it widely applicable in various clinical settings [39]. Participants rate how often they have experienced anxiety symptoms over the past two weeks using a 4-point Likert scale ranging from 0 to 3. The total score ranges from 0 to 21, with the following classifications: a score of 0–4 points indicates no or minimal anxiety, 5–9 points indicates mild anxiety, 10–14 points indicates moderate anxiety, and 15–21 points indicates severe anxiety. While mild anxiety does not significantly affect daily life, scores of 10 or higher may indicate difficulties in social functioning, work performance, and personal life. In this study, the version validated by Seo & Park [40] was used, with a Cronbach’s α of 0.915. Cronbach’s α for this study was 0.90.

#### 2.4.4. Perceived Stress Scale (PSS)

The PSS-10 is an instrument developed by Cohen et al. [41] to assess an individual’s perception of their stress levels over the past month. It consists of 10 items rated on a 5-point Likert scale, ranging from 0 (never) to 4 (very often). The total score ranges from 0 to 40, with 0–13 indicating low stress, 14–26 indicating moderate stress, and 27 and above indicating high stress. In this study, the Korean version adapted by Lee et al. [42] was used, with a Cronbach’s α of 0.82. Cronbach’s α for this study was 0.64.

#### 2.4.5. Korean Version of the Insomnia Severity Index (ISI-K)

The ISI-K consists of 7 items that assess difficulties in falling asleep, staying asleep, waking up too early, satisfaction with sleep patterns, functional impairment in daily life, and the impact of sleep problems on quality of life and distress over the past two weeks. Each item is rated on a scale from 0 to 4, and the severity of insomnia is measured by the total score obtained by summing the scores of all items. A score of 15 or above is considered indicative of clinically significant insomnia. This tool was originally developed by Morin et al. [43] to systematically evaluate insomnia. In Korea, its reliability and validity were verified by Cho et al. [44], with a Cronbach’s α of 0.92. Cronbach’s α for this study was 0.86.

### 2.5. Statistical Analysis

Data analysis was conducted using SPSS 23 and RStudio (version 4.4.1). After examining the means and distributions of each scale for all participants, a mixed-design ANCOVA was performed, with sex and age and pre-score as covariates. To address initial group differences observed in baseline scores, pre-intervention scores were included as covariates. In addition, since significant group differences were found in sex and age, these variables were also included as covariates to reduce potential confounding effects on the outcome measures. In the analysis, group (intervention vs. control) was set as the between-subjects factor, and time (weeks 1, 2, 4, and 8) was set as the within-subjects (repeated-measures) factor.

PM2.5 concentrations were treated as a fixed value by group × time based on the average PM2.5 level over the 7-day period during which each session was held. This is because it was not possible to measure real-time individual exposure to PM2.5, and the influence of external environmental factors could not be completely excluded in the locations where the program was conducted. Therefore, PM2.5 was not included as a covariate but was incorporated into the analysis as an interaction term (group × PM2.5).

### 2.6. Ethics Statement

This study was conducted in accordance with the Declaration of Helsinki and was approved by the Institutional Review Board of Dankook University (DKU 2024-07-002). Voluntary informed consent was obtained from all participants and their guardians after explaining the purpose and procedures of the study. Participants were also informed that they could withdraw from the study at any time without any penalty.

## 3. Results

### 3.1. Participant Demographics

A total of 95 participants were included in the final analysis. The control group consisted of 51 students (16 males, 31.4%; 35 females, 68.6%), while the intervention group comprised 44 students (25 males, 56.8%; 19 females, 43.2%). There was a significant difference in gender distribution between the two groups (χ^2^ = 6.23, *p* = 0.01). Additionally, the mean age of the control group was 11.77 years (±0.42), while the intervention group had a mean age of 9.90 years (±0.57), indicating a statistically significant age difference between the groups (t = −17.81, *p* < 0.001). The baseline characteristics are summarized in Table 1.

### 3.2. Differences in General Knowledge About Fine Dust

The intervention group showed significant post-program improvements in knowledge related to fine dust, including the definition of fine dust, the fine dust forecasting system, behaviors to avoid exposure, the need to check concentrations, the health benefits of monitoring concentrations, and the need for indoor ventilation. Improvements were also observed in preventive actions such as checking dust levels. In the control group, significant improvements were found only in knowledge of the definition of fine dust and the forecasting system, with no statistically significant changes in most other items. Detailed results, including item-level response rates and significance tests, are presented in Table 2.

### 3.3. Differences in Depression, Anxiety, Stress, and Insomnia Between the Two Groups

Anxiety scores showed a significant decrease over time in the intervention group (F = 4.46, *p* = 0.01, partial η^2^ = 0.09). Specifically, anxiety scores in the intervention group decreased gradually from 0.79 ± 0.77 at the first measurement to 0.60 ± 0.72 at the second measurement, to 0.47 ± 0.57 at the third measurement, and 0.23 ± 0.57 at the fourth measurement. In contrast, the anxiety scores of the control group remained almost unchanged, ranging from 0.23 ± 0.48 at the first measurement to 0.26 ± 0.54 at the second measurement, 0.31 ± 0.66 at the third measurement, and 0.30 ± 0.62 at the fourth measurement, and the time effect was not significant (F = 0.07, *p* = 0.98).

The difference between groups was significant (F = 10.74, *p* = 0.00, partial η^2^ = 0.03), and the group × time interaction effect was also significant (F = 6.08, *p* = 0.01, partial η^2^ = 0.04). This suggests that anxiety scores decreased significantly only in the intervention group. However, the PM2.5 × group effect was not significant (F = 0.08, *p* = 0.78).

For depression scores, the intervention group showed a significant decrease over time (F = 4.47, *p* = 0.01, partial η^2^ = 0.06). No significant changes over time were observed in either the intervention or control groups. The intervention group showed a continuous decrease from 1.52 ± 1.66 at baseline to 1.00 ± 1.14 at follow-up 2, 0.97 ± 1.03 at follow-up 3, and 0.83 ± 1.15 at follow-up 4. Although a gradual decrease was observed, it was not statistically significant (F = 1.64, *p* = 0.18, partial η^2^ = 0.04). The control group also showed a slight decrease from 0.79 ± 1.03 at the first measurement to 0.58 ± 0.85 at the second measurement, 0.70 ± 1.01 at the third measurement, and 0.60 ± 1.12 at the fourth measurement, but the change was not significant (F = 0.76, *p* = 0.52). There were no significant differences between groups (F = 0.27, *p* = 0.60), and neither the group × time interaction effect (F = 2.36, *p* = 0.13) nor the PM2.5 × group effect (F = 0.34, *p* = 0.56) were significant. This suggests that, even though the decrease in depression scores in the intervention group was significant, the magnitude of the change was not statistically significant when compared to the control group.

In terms of stress scores, no significant changes over time were observed in either the intervention or control groups. The stress scores in the intervention group decreased slightly from 10.72 ± 4.82 at baseline to 8.93 ± 5.37 at the second measurement, and 8.84 ± 5.58 at the third measurement, and 9.3 ± 6.51 at the fourth measurement, a slight increase (F = 1.40, *p* = 0.25, partial η^2^ = 0.03). The control group also showed a similar pattern, with stress scores decreasing from 11.05 ± 5.44 at the first measurement to 10.30 ± 5.40 at the second measurement, 9.46 ± 6.46 at the third measurement, and 9.36 ± 6.17 at the fourth measurement (F = 1.75, *p* = 0.16). The group effect (F = 1.68, *p* = 0.20), group × time interaction effect (F = 0.02, *p* = 0.90), and PM2.5 × group effect (F = 0.01, *p* = 0.94) were all not significant.

For insomnia scores, no significant changes over time were observed in either the intervention or control groups. In the intervention group, the scores decreased gradually from 2.83 ± 2.33 at baseline to 2.10 ± 2.41 at the second visit, 1.94 ± 2.46 at the third visit, and 2.17 ± 2.41 at the fourth visit, but this decrease was not statistically significant (F = 1.59, *p* = 0.19, partial η^2^ = 0.03). In the control group, the mean score decreased from 1.31 ± 1.30 at baseline to 1.21 ± 1.81 at the second visit, 1.44 ± 2.01 at the third visit, and 2.83 ± 2.33 at the fourth visit, showing a pattern of alternating increases and decreases (F = 0.32, *p* = 0.81). Neither the group effect (F = 0.38, *p* = 0.54), the group × time interaction (F = 0.54, *p* = 0.46), nor the PM2.5 × group effect (F = 0.01, *p* = 0.91) were statistically significant.

In summary, only in anxiety did the intervention group show a significant gradual decrease over time, along with significant group differences and interaction effects, while no significant changes were observed in depression, stress, or insomnia. A significant gradual decrease over time was observed in the intervention group for anxiety and depression, and in anxiety, group differences and group × time interaction effects were also significant. However, for depression, the decrease was significant only within the intervention group, and the comparison with the control group was not significant, making it difficult to confirm the intervention effect. For stress and insomnia, neither the time effect, group effect, nor interaction effect was significant. The results for depression, anxiety, stress, and insomnia in both the intervention and control groups are presented in Table 3.

## 4. Discussion and Conclusions

This study implemented a CBT-based fine dust education program for elementary school students living near a coal-fired power plant to examine its effects on general knowledge about fine dust and mental health.

In the intervention group, there were statistically significant improvements in knowledge and behavioral change across six areas: understanding the definition of fine dust, awareness of the fine dust forecasting system, recognition of preventive behaviors, checking PM2.5 levels, awareness of the health benefits of monitoring fine dust, and understanding the need for indoor ventilation on days with high PM2.5 levels. Notably, the rate of understanding the definition of fine dust increased from 33.3% before the intervention to 75.0% afterward, and awareness of forecasting systems rose from 19.0% to 81.8%. These increases were even more pronounced than those observed in the control group (definition awareness: from 58.0% to 80.4%; forecasting system awareness: from 56.9% to 80.4%). These findings suggest that the CBT-based intervention program may have effectively enhanced children’s cognitive understanding and behavioral changes regarding fine dust through learner-centered activities, peer interaction, and the use of visual materials.

The results are also consistent with a previous study that evaluated the effectiveness of educational interventions based on the HBM, which sought to improve perceived susceptibility, perceived severity, perceived benefits, and self-efficacy [45]. The program promoted preventive behaviors among children by increasing their awareness of the risks and severity of fine dust, emphasizing the benefits of preventive behaviors, enhancing self-efficacy through practical strategies, and providing behavioral cues through peer interaction and audiovisual materials. In particular, the activity-based participatory learning likely functioned as a cue to action (a factor that encourages engagement in preventive behaviors) as presented in the HBM [46]. Meanwhile, the interaction effect between PM2.5 and group was not statistically significant in either the intervention or control group. This suggests that PM2.5, an external environmental factor, did not directly influence the psychological indicators of the two groups. Instead, it implies that the structural characteristics and content of the intervention program may have had a more significant impact on psychological changes.

When analyzing the impact of the program on mental health (depression, anxiety, stress, sleep problems, etc.), for stress and sleep problems, neither the between-group difference, change over time, or group × time interaction effect was statistically significant. For depression, there was a significant decrease in scores over time in the intervention group, but the between-group difference and interaction effects were not significant, making it difficult to conclusively interpret the effect of the intervention compared to the control group. For anxiety, both the group effect and the group × time interaction effect were significant, and the gradual decrease over time in the intervention group was also statistically significant. This suggests that the cognitive behavioral therapy (CBT)-based particulate matter education program was effective in reducing children’s anxiety, with the main effect of the intervention being specific to anxiety. This is consistent with previous studies showing that cognitive behavioral therapy-based approaches reduce anxiety [47], and while some within-group improvement was observed for depression, there were no significant differences in the between-group comparisons, which may be interpreted as inconsistent with previous studies showing that cognitive behavioral therapy-based approaches reduce depression [48,49], stress [50,51], and sleep problems [52,53].

CBT-based approaches can improve emotional regulation in various ways, and within the same intervention, the mechanisms for enhancing emotional regulation may differ from one person to another [54]. For some children, participating in specific tasks may have significantly contributed to emotional regulation, while for others, relaxation training may have been the key element. Particularly, the use of relaxation techniques in CBT can help adolescents recognize and differentiate between physiological arousal and tension levels, allowing them to implement strategies to reduce arousal [55]. The program included strategies such as deep breathing and muscle relaxation, which may have contributed to lower physiological arousal levels.

On the other hand, the reason the program had a limited effect on improving mental health areas (depression, stress, and sleep problems) other than anxiety may be because the program was structured around improving awareness and practical actions related to fine dust, rather than directly addressing mental health problems. This limited effect on depressive and stress symptoms aligns with recent meta-analytic findings showing that school-based programs are generally more effective when they are targeted toward at-risk groups, rather than universally delivered, with universal interventions yielding smaller or short-lived effects in depression, anxiety, and stress outcomes [56].

As a result, the program was effective in improving children’s general knowledge related to fine dust, and had a positive effect on mental health indicators such as anxiety. Consequently, the program was found to be more effective in enhancing children’s general knowledge about fine dust and had a positive impact, specifically on anxiety levels.

However, this study does have some limitations. First, the sample size was relatively small, with only 95 participants recruited, making it difficult to generalize the findings. Second, since the research variables were measured using self-report questionnaires, respondents may not have reported themselves honestly. Third, both the intervention group and the control group were selected from schools located in the midwestern region of Korea, which is vulnerable to fine dust; however, it is difficult to consider this as reflecting the characteristics of various communities. Fourth, epidemiological variables such as gender and age were not directly controlled at the sampling stage, although they were included as covariates in the ANCOVA models to minimize their confounding effects. As a result, various participant factors may have influenced the program’s effectiveness. Despite these limitations, this study holds practical significance as it examined the effects of a CBT-based intervention program for children vulnerable to fine dust. The program effectively enhanced children’s general knowledge about fine dust and demonstrated some positive psychological effects, such as reduced anxiety. This suggests that it could provide valuable foundational data for the development of future fine dust education programs. Future research should include follow-up programs with longer and more intensive interventions, control for confounding variables such as temperament and family environment, and conduct larger-scale validation and assessment of treatment effectiveness.

## Figures and Tables

**Table 1:** Epidemiological characteristics of the intervention and comparison groups.

Variables	Intervention (n = 51)	Control (n = 44)	t/χ^2^	*p* Value
Age	11.77 ± 0.42	9.90 ± 0.57	−17.81	0.00
Sex			6.23	0.01
Male	16 (31.4)	25 (56.8)		
Female	35 (68.6)	19 (43.2)		

Data represent mean ± standard deviation, by independent *t*-test, or n (%), by chi-square test.

**Table 2:** Changes in general knowledge about fine dust (%P) in the intervention and control groups.

Survey Item	Intervention Group				Control Group				Difference Pre and Post (%)	
	Pre	Post	χ^2^	*p*-Value	Pre	Post	χ^2^	*p*-Value	Intervention	Control
I understand the definition of the dust (PM2.5)	33.3	75.0	15.05 *	<0.00	58.0	80.4	5.95 *	0.02	41.7	22.4
I am familiar with the fine dust forecasting system	19.0	81.8	33.89 *	<0.00	56.9	80.4	6.56 *	0.01	62.8	23.5
I know how to avoid fine dust	90.5	100.0	4.40 *	0.04	98.0	96.1	0.34	0.56	9.5	−1.9
I am aware of the health risks associated with fine dust exposure	97.6	97.7	0.00	0.97	98.0	94.1	1.04	0.31	0.1	−3.9
I believe I can reduce my exposure to fine dust through my own efforts	73.8	84.1	1.37	0.24	86.3	84.3	0.08	0.78	10.3	−2
I believe I should avoid outdoor activities on days with high PM2.5 levels	86.4	90.9	0.45	0.50	80.4	82.4	0.07	0.80	4.5	2
I refrain from outdoor activities on days with high PM2.5 levels (habitual behavior)	59.1	72.7	1.82	0.18	66.7	66.7	0.00	1.00	13.6	0
Avoiding outdoor activities on days with high PM2.5 levels effectively protects my health	88.4	95.5	1.47	0.23	84.3	82.4	0.07	0.79	7.1	−1.9
I believe I should check PM2.5 levels	84.1	97.7	4.95 *	0.03	72.5	84.3	2.09	0.15	13.6	11.8
I regularly check PM2.5 levels	45.5	70.5	5.64 *	0.02	56.9	62.7	0.37	0.55	25	5.8
Checking PM2.5 levels is effective in protecting my health	81.8	95.5	4.06 *	0.04	80.0	82.4	0.09	0.76	13.7	2.4
I believe indoor ventilation is necessary on days with high PM2.5 levels	34.1	56.8	4.58 *	0.03	37.3	52.9	2.53	0.11	22.7	15.6
I ventilate indoors on days with high PM2.5 levels (habitual behavior)	40.9	54.5	1.64	0.20	31.4	49.0	3.30	0.07	13.6	17.6
Ventilating indoors on days with high PM2.5 levels effectively protects my health	59.1	70.5	1.25	0.27	64.7	74.5	1.16	0.28	11.4	9.8
I believe I should wear a mask on days high PM2.5 levels	93.2	88.6	0.55	0.46	90.2	94.1	0.54	0.46	−4.6	3.9
I wear a mask on days with high PM2.5 levels (habitual behavior)	65.1	47.7	2.67	0.10	72.5	68.6	0.19	0.66	−17.4	−3.9
Wearing a mask on days with high PM2.5 levels effectively protects my health	88.6	95.3	1.32	0.25	92.0	94.1	0.17	0.68	6.7	−2.1

Data represent mean ± standard deviation n (%) by chi-square test, * *p* < 0.05.

**Table 3:** Scores for depression, anxiety, stress, and insomnia of the intervention and control groups. Results of mixed-design ANCOVA on psychological scales, adjusting for sex and age and baseline scores. PM2.5 was included as a fixed group × time interaction variable.

Variables	Group	1st (Mean ± SD)	2nd (Mean ± SD)	3rd (Mean ± SD)	4th (Mean ± SD)	Time F (*p*) /Partial η^2^	Group F (*p*) /Partial η^2^	Group × Time F (*p*) /Partial η^2^	Group × PM2.5 F (*p*) /Partial η^2^
Depression (PHQ)	Intervention	1.52 ± 1.66	1.00 ± 1.14	0.97 ± 1.03	0.83 ± 1.15	4.46 (0.01) * /0.06	0.27 (0.60) /0.02	2.36 (0.13) /0.01	0.34 (0.56) /0.00
	Control	0.79 ± 1.03	0.58 ± 0.85	0.70 ± 1.01	0.60 ± 1.12	0.76 (0.52) /0.01			
Anxiety (GAD)	Intervention	0.79 ± 0.77	0.60 ± 0.72	0.47 ± 0.57	0.23 ± 0.57	4.47 (0.01) * /0.09	10.74 (0.00) * /0.03	6.08 (0.01) * /0.04	0.08 (0.78) /0.00
	Control	0.23 ± 0.48	0.26 ± 0.54	0.31 ± 0.66	0.30 ± 0.62	0.07 (0.98) /0.00			
Stress (PSS)	Intervention	10.72 ± 4.82	8.93 ± 5.37	8.84 ± 5.58	9.3 ± 6.51	1.40 (0.25) /0.03	1.68 (0.20) /0.01	0.02 (0.90) /0.00	0.01 (0.94) /0.00
	Control	11.05 ± 5.44	10.30 ± 5.40	9.46 ± 6.46	9.36 ± 6.17	1.75 (0.16) /0.02			
Insomnia (ISI)	Intervention	2.83 ± 2.33	2.10 ± 2.41	1.94 ± 2.46	2.17 ± 2.41	1.59 (0.19) /0.03	0.38 (0.54) /0.02	0.54 (0.46) /0.00	0.01 (0.91) /0.00
	Control	1.31 ± 1.30	1.21 ± 1.81	1.44 ± 2.01	2.83 ± 2.33	0.32 (0.81) /0.00			

Data represent mean ± standard deviation. PHQ: Patient Health Questionnaire, GAD: Generalized Anxiety Disorder Assessment, PSS: Perceived Stress Scale, ISI: Insomnia Severity Index. Data represent F-values and partial η^2^ for each main and interaction effect. * *p* < 0.05.

## Data Availability

The datasets generated and analyzed during the current study are available from the corresponding authors on reasonable request.
